# Central Arterial Stiffness and Cognitive Function in Patients with Mild Cognitive Impairment

**DOI:** 10.1002/alz70857_099806

**Published:** 2025-12-24

**Authors:** Aryan Verma, Junyeon Won, Tsubasa Tomoto, Munro Cullum, Rong Zhang

**Affiliations:** ^1^ University of Texas at Dallas, Richardson, TX, USA; ^2^ Institute of Exercise and Environmental Medicine, Texas Health Presbyterian Hospital Dallas, Dallas, TX, USA; ^3^ University of Texas Southwestern Medical Center, Dallas, TX, USA

## Abstract

**Background:**

Elevated central arterial stiffness is a risk factor for amnestic mild cognitive impairment (aMCI) and dementia, but the pathophysiological mechanisms underlying the relationship between vascular health and cognitive decline in patients with aMCI remain unclear. We examined the cross‐sectional relationship between central arterial stiffness, brain default mode network functional connectivity (DMN‐FC), and cognitive function in patients with aMCI.

**Method:**

Participants aged 55‐80 years, either cognitively normal or diagnosed with aMCI, and without major neurological, vascular, metabolic, or psychiatric diseases, were recruited. Data was analyzed from 21 cognitively normal (age 65.9±7.0) and 48 adults with aMCI (age 64.1±5.9). Central arterial stiffness was measured using carotid‐femoral pulse wave velocity (cfPWV) and carotid β‐stiffness via applanation tonometry and ultrasonography. DMN‐FC was assessed with resting‐state fMRI and seed‐based correlation analysis, using the posterior cingulate cortex (PCC) as the seed. Episodic memory and executive function were evaluated with California Verbal Learning Test (CVLT) and Wisconsin Card Sorting Test (WCST). Data was analyzed using multiple variable linear regression and mediation analysis.

**Result:**

Carotid β‐stiffness index was slightly higher in aMCI patients (8.9±2.1) compared to cognitively normal older adults (8.0±1.7), but the difference was not statistically significant. Higher carotid β‐stiffness index was associated with higher DMN‐FC between the PCC and right precentral gyrus (PcG) in the cognitively normal (B=0.02, r^2^ = 0.373, *p* = 0.003) and aMCI group (r^2^ = 0.260, *p* = 0.0002). Higher DMN‐FC between the PCC and right PcG was associated with worse CVLT performance in the aMCI group (B=‐27.10, *p* = 0.012, 95%CI = ‐47.91, ‐6.30). Higher carotid β‐stiffness index was associated with worse CVLT performance (B=‐1.55, *p* = 0.045, 95%CI=‐3.08, ‐0.03) and worse WCST performance (B=‐2.62, *p* = 0.068, 95%CI = ‐5.45, 0.20) in the aMCI group. Higher DMN‐FC between the PCC and right PcG mediated the association between higher carotid β‐stiffness index and worse CVLT performance in the aMCI group.

**Conclusion:**

Our findings suggest that elevated central arterial stiffness may impair cognitive performance in patients with aMCI through alterations in DMN functional connectivity, potentially reflecting compensatory mechanisms. These findings underscore the importance of addressing vascular health as part of efforts to prevent or delay cognitive decline in aging populations, particular those at risk of dementia.

